# Prevalence and Characteristics Associated with Malnutrition at Hospitalization among Patients with Acquired Immunodeficiency Syndrome in Brazil

**DOI:** 10.1371/journal.pone.0048717

**Published:** 2012-11-07

**Authors:** Carine S. Andrade, Rosângela P. Jesus, Taciana B. Andrade, Neyara S. Oliveira, Scott A. Nabity, Guilherme S. Ribeiro

**Affiliations:** 1 School of Nutrition, Federal University of Bahia, Salvador, Bahia, Brazil; 2 School of Medicine, Duke University, Durham, North Carolina, United States of America; 3 Gonçalo Moniz Research Center, Oswaldo Cruz Foundation, Brazilian Ministry of Health, Salvador, Bahia, Brazil; 4 Institute of Collective Health, Federal University of Bahia, Salvador, Bahia, Brazil; Asociacion Civil Impacta Salud y Educacion, Peru

## Abstract

**Background:**

Brazil’s National STD/AIDS Program is considered a model of success worldwide. However, AIDS-associated malnutrition continues in subgroups of Brazilian patients despite access to free highly active antiretroviral therapy (HAART). We aimed to identify the prevalence of malnutrition and associated factors among patients hospitalized with AIDS.

**Methods:**

We conducted a cross-sectional nutritional assessment among 127 adults hospitalized with AIDS in Brazil’s third largest city. Using anthropometric measurements, we determined the prevalence of malnutrition (body mass index <18.5 kg/m2) at hospitalization. Prevalence ratios of malnutrition by demographic, socioeconomic, and clinical conditions were estimated using log-binomial regression.

**Results:**

One-third of participants were first informed of their HIV disease during the current hospitalization and recent treatment interruption was common (71%) among those on HAART. Forty-three percent were malnourished and 35% had severe weight loss at admission. Patient characteristics independently associated with malnutrition were older age (2% increased prevalence for each year; 95% confidence interval [CI] 0–4%) and very low daily *per capita* household income. Living on <USD 2.00, USD 2.00–4.99 or USD 5.00–9.99 increased the prevalence of malnutrition by 2.01 (95% CI 1.06–3.81), 1.75 (95% CI 0.92–3.35) and 1.42 (95% CI 0.76–2.65) times, respectively, compared to ≥USD 10.00 per day. Chronic diarrhea was marginally associated with malnutrition (RR 1.42; 95% CI 0.99–2.04). Overall, 16% of the patients died during hospitalization. We observed a trend toward higher in-hospital case fatality among malnourished patients (22% vs. 12% for patients with and without malnutrition, respectively; chi square P = 0.14).

**Conclusions:**

Unacceptably high rates of malnutrition persist in Brazilians hospitalized with AIDS and our results reinforce the importance of nutritional evaluations in these patients. Improved early testing and treatment adherence strategies may continue to help reduce AIDS-related morbidity and mortality in Brazil, yet novel interventions to disrupt the cycle of poverty, HIV, and malnutrition are also urgently needed.

## Introduction

Weight loss and malnutrition are among the most common clinical findings observed in patients with untreated acquired immunodeficiency syndrome (AIDS) [1]. Malnutrition in these patients has multiple determinants, including reduction in food intake, nutrient malabsorption, and increased energy expenditure due to the hypercatabolic state caused by the human immunodeficiency virus (HIV) infection itself and opportunistic diseases [2], [3]. In turn, malnutrition further compromises the immune system and has been consistently associated with increased risk of death [4]–[7].

Introduction of highly active antiretroviral therapy (HAART) has dramatically changed the course of HIV infection in countries that prioritized its distribution. Brazil was an early adopter of freely available HAART as part of the National STD/AIDS Program and is recognized worldwide for operating at the forefront on AIDS [8]. HAART sustainably suppresses viral replication, allowing recovery of the immune system. As a consequence, AIDS-associated mortality and morbidity declined after the widespread introduction of HAART [9] and mortality rates for HIV-infected individuals with high CD4 cell counts and HAART use are similar to the general population [10].

Most of the nutritional concerns in AIDS care in countries where HAART is widely available are now related to metabolic alterations associated with HAART, which predispose patients to cardiovascular [11] and other chronic complications [12], [13]. However, even in the HAART era, weight loss and malnutrition remain common problems for certain HIV infected subgroups, such as those diagnosed late in the course of the infection and those with failed or non-adherent antiretroviral regimens [14]. To draw attention to the importance of proper nutritional care for such vulnerable patients, we aimed to quantify the prevalence of malnutrition in patients with AIDS consecutively admitted at the reference hospital for infectious diseases in Salvador, Brazil and to investigate patient characteristics associated with malnutrition at hospital admission.

## Methods

### Study Design and Participants

We conducted a cross-sectional study at the reference hospital for infectious diseases in Salvador, the third largest city in Brazil (2,480,790 inhabitants) [15], between June 2009 and March 2010. The 101-bed state hospital is one of three public health institutions providing specialized inpatient care for patients with AIDS in Salvador and it accounted for 32% of citywide AIDS hospitalizations during the study period [16]. Using an estimated prevalence of malnutrition of 50%, we determined our target sample size (n = 118) to achieve a precision of +/−8% around the measured prevalence of malnutrition. This figure was based on the expected number of AIDS-related hospitalizations in persons 20 to 59 years of age in Salvador in 2008 [16].

We recruited participants by reviewing hospital registries five days a week and consecutively enrolling all patients from 20 to 59 years of age who: 1) were admitted to the hospital with a known diagnosis of AIDS, or 2) demonstrated serological evidence of HIV infection with a rapid test (DPP HIV 1/2; Bio-Manguinhos, Rio de Janeiro, Brazil) and met the U.S. Centers for Disease Control and Prevention (CDC) definition for AIDS in the first seven days of hospitalization [17]. Patients were ineligible for study entry if they required urgent intensive care support or if they were cognitively impaired and unaccompanied by a legal representative to provide informed consent. Patients with repeated hospitalizations during the study period were enrolled in the study only once. Patients diagnosed with AIDS after the seventh day of hospitalization were also ineligible for study entry because nutritional evaluation at that time could be unrepresentative of nutritional status at hospital admission.

### Data Collection

We interviewed patients and reviewed charts using standardized forms to obtain data on demographics, socioeconomic indicators, and clinical history, including current or prior HAART use. By chart review, the study team confirmed the serological diagnosis of HIV infection, collected initial hemoglobin and albumin levels upon hospitalization, and obtained the most recent CD4 cell count and HIV load performed at the state public health reference laboratory. Lastly, we systematically identified the clinical conditions associated with the decision to hospitalize, and we assessed length of hospitalization, intensive care unit admission, and death during hospitalization as clinical outcomes.

### Nutritional Evaluation

Prior to study initiation, the study team was trained to standardize the anthropometric exam. We evaluated nutritional status during the first week of hospitalization. For patients that were not restricted to bed, we directly measured weight in kilograms using a calibrated portable digital balance (Filizola; São Paulo, Brazil) with capacity up to 150 kg and precision of 100 g and we directly measured height in centimeters using a 205 cm stadiometer (Seca Leicester; Hamburg, Germany). We also measured mid-upper arm circumference and tricipital skinfold of the non-dominant arm, according to the procedures described by Lohman et al. [18]. For bed-restricted patients, we obtained knee height, calf circumference, and non-dominant subscapular skinfold and mid-upper arm circumference measurements as previously described [19], [20] and we estimated weight and height using the formulas of Chumlea et al. [20], [21]. In addition, we measured tricipital skinfold of the non-dominant arm, according to previously described procedures [19].

To measure circumferences, skinfold thickness and knee height, we used an inelastic measuring tape of 1 mm precision, adipometer skinfold calipers (Lange Beta Technology Inc.; Santa Cruz, CA, USA) and an anthropometer (Fami Itá Products; São Caetano do Sul, Brazil), respectively. We measured skinfold thickness in duplicate from which we calculated a mean skinfold thickness. When the difference between the observed skinfold thickness was greater than 1 mm, we performed a third measurement and calculated the mean between the two closest measurements.

We calculated body mass index (BMI) by dividing patient weight in kilograms by the square of patient height in meters and we applied the World Health Organization criteria of BMI <18.5 kg/m^2^ to classify patients as malnourished [22]. We estimated the percentage of body weight loss based on the weight at hospital admission and the patient’s self-reported weight of six months prior to this hospitalization. The mid-upper arm circumference and the tricipital skinfold thickness were used to calculate the mid-upper arm muscle area with a correction for the bone area [23].

### Statistical Methods

We analyzed data using Epi Info version 3.4.3 (U.S. CDC; Atlanta, USA) and SAS 9.1.3 (SAS Institute Inc.; Cary, USA). Patient characteristics were described using frequencies for categorical variables and medians and interquartile ranges for continuous variables. For two patients with undetectable levels of plasma HIV RNA, we assigned the HIV viral load as log_10_ 1.70 copies/mL (50 copies/mL). We categorized CD4 counts as above or below 200 cells/mm^3^. We also categorized the time since patients’ first knowledge of their HIV disease in relation to the current hospitalization as occurring at hospitalization, within 2 years (representing an opportunity for recent initiation of antiviral therapy), 3–10 years prior (intermediate-term survivors), or ≥11 years prior (long-term survivors). Lastly, we converted monthly household income from the Brazilian currency (real) to the United States dollar (USD) using the average exchange rate of 0.5485 for the study period and stratified the patients according to their daily per capita household income as USD <2.00, 2.00–4.99, 5.00–9.99 and ≥10.00.

We considered weight loss of 10.1% to 20.0% to be moderate and weight loss >20.0% to be severe. From tricipital skinfold thickness measurements we estimated body fat composition [19] and from the mid-upper arm muscle area with a correction for the bone area we estimated lean body mass according to the formula developed by Heymsfield [24] and adapted by Gibson [25]. Subsequently, we compared measurements of tricipital skinfold thickness and corrected mid-upper arm muscle area to population norms and classified them as normal (>15^th^ percentile), mild to moderate depletion (5^th^–15^th^ percentiles) or severe depletion (<5^th^ percentile) [26].

We investigated demographic, socioeconomic and clinical characteristics association with malnutrition (BMI<18.5 kg/m^2^) at hospital admission with exploratory analyses. We compared proportions using the Chi-square test or the Fisher’s exact test and we compared the median values of non-normally distributed continuous variables using the nonparametric Wilcoxon-Mann-Whitney test. Unadjusted and adjusted prevalence ratios (PR) and 95% confidence intervals (95% CI) were estimated using log-binomial regression models from univariate and multivariable analyses, respectively [27]. Backward elimination analyses included those variables associated with malnutrition (two-tailed test, α = 0.10) and those thought to be clinically relevant (i.e., years of formal education, employment status, time from HIV disease to current hospitalization, CD4 count <200 cells/mm^3^, and diagnosis of pulmonary tuberculosis at hospitalization) to adjust for confounding. From this process, we chose the final model with the best fit according to Akaike’s information criterion (AIC). Finally, we calculated the risk ratio and 95% confidence interval for death in relation to malnutrition.

### Ethics Statement

The research protocol was approved by the Institutional Review Boards of Hospital Couto Maia and the School of Nutrition, Federal University of Bahia, both in Salvador, Brazil. All participants or their legal representative (first degree relative or spouse) in the case of cognitive impairment agreed to participation in this research by signing a written informed consent. The data were analyzed anonymously.

## Results

### Patient Recruitment

During the ten-month study period, 185 unique patients were hospitalized with AIDS at the study hospital. Of these, 31 were not eligible for enrollment due to cognitive impairment in the absence of legal representative to provide informed consent (15 patients), immediate requirement of intensive care support (9) and diagnosis of AIDS after the seventh day of hospitalization (7). Of the 154 eligible patients, 127 (82%) were included in the study, as 17 (11%) refused participation and 10 (6%) were identified by the study team ≥7 days after hospital admission.

### Patient Characteristics

Of the study participants, 78 (61%) were male, 120 (94%) were black or mixed race, and the median age was 36 years (interquartile range [IQR] 30–44) (Table 1). The patient population reported low levels of socioeconomic status, as 28 (22%) were living in absolute poverty with a *per capita* household income of less than USD 2.00 a day and 103 (81%) were living on less than USD 10.00 a day. Overall, 35 (28%) participants received direct cash payments from the Brazilian government as part of a national program (*bolsa familia*) to reduce severe poverty and food insecurity.

**Table 1:** Sociodemographic and clinical characteristics of patients hospitalized with AIDS.

Category	Characteristic		n	Number (%) or median [IQR] (N = 127)
Demographic	Male sex		127	78 (61)
	Age (years)		127	36 [30]–[44]
	Race	Black	127	68 (53)
		Mixed		52 (41)
		White		7 (6)
Socioeconomic	Formal education (years)		127	7 [5]–[11]
	Formally employed		127	20 (16)
	Participant of cash payments program*		127	35 (28)
	*Per capita* household income (USD/day)	< $2.00	127	28 (22)
		$2.00–$4.99		41 (34)
		$5.00–$9.99		34 (28)
		≥ $10.00		24 (20)
Clinical	Time from HIV disease to current hospitalization†	At hospitalization‡	125	40 (32)
		≤2 years prior		36 (29)
		3–10 years prior		36 (29)
		≥11 years prior		13 (10)
	Prior HIV-related hospitalizations		85§	59 (69%)
	HAART¶		85§	58 (68)
	CD4 count (cells/mm^3^)		100	104 [43–215]
	HIV load (log_10_ copies/mL)		94	4.92 [4.00–5.33]
Outcome	Days of hospitalization		118	17 [10]–[35]
	ICU admission		118	14 (12)
	Death during hospitalization		118	19 (16)

* Self-reported participant of a direct cash payments program (*bolsa família*) from the Brazilian government as part of a national effort to reduce severe poverty and food insecurity.

† Represents the length of time the patient was aware of diagnosis of HIV disease prior to current hospitalization.

‡ Diagnosis made at current hospitalization.

§ Denominator includes only those 85 patients with knowledge of their HIV disease prior to current hospitalization.

¶ Includes self-reported current or former HAART use.

IQR = interquartile range.

USD = United States dollar.

HAART = highly active antiretroviral therapy.

ICU = intensive care unit.

Of 125 patients with available data on the timing of HIV disease diagnosis, 40 (32%) were first informed of their HIV disease during the current hospitalization, 36 (29%) within 2 years, 36 (29%) from 3–10 years, and 13 (10%) more than 10 years prior to the current hospitalization. Of the 85 patients who were already aware of their HIV infection at admission, 59 (69%) recalled at least one prior HIV-related hospitalization (median 2 [IQR 2–4] prior hospitalizations) and 58 (68%) reported current or prior HAART use (Table 1). Among HAART users, 41 (71%) reported an interruption in therapy within the 6 months prior to hospitalization.

The CD4 cell count was lower than 200 cells/mm^3^ for 73 of the 100 patients with available CD4 results (median 104 [IQR: 43–215] cells/mm^3^, Table 1) and HIV loads were generally high (median log_10_ viral load 4.92 [IQR 4.00–5.33]). Nonetheless, patients who had previously used HAART presented with higher CD4 counts (median of 160 cells/mm^3^ for HAART users vs. 83 cells/mm^3^ for never users; Wilcoxon P = 0.03) and lower log_10_ HIV load (median of 4.51 log_10_ copies/mL for HAART users vs. 5.07 log_10_ copies/mL for never users; Wilcoxon P = 0.003). These findings were maintained when excluding patients informed of their HIV disease at this hospitalization (data not shown).

The most frequent medical conditions associated with hospitalization included oroesophageal candidiasis (61 patients, 48%), chronic diarrhea (>30 days) (52, 41%), pulmonary tuberculosis (34, 27%), neurotoxoplasmosis (25, 20%), meningitis (17, 13%), pneumonia (17, 13%) and extrapulmonary tuberculosis (14, 11%), and a majority (68, 53%) had multiple medical conditions leading to hospitalization. Among patients with multiple diagnoses at admission, oroesophageal candidiasis (49, 72%) and diarrhea (38, 56%) were most frequently identified. Participants were hospitalized for a median duration of 16 days [IQR 9–34] (Table 1). Of the 127 patients we enrolled, nine were transferred to another hospital and clinical outcomes could not be evaluated. For the remaining 118 patients, 14 (12%) required intensive care and 19 (16%) died during hospitalization (Table 1).

### Nutritional Status

We performed the nutritional evaluation within 72 hours of hospital admission for the majority (104 patients, 82%) of those enrolled. Table 2 shows that nutritional characteristics were similar between patients who had height and weight estimated due to bed restriction (29, 23%) and those who underwent direct measurement (98, 77%). For the 102 patients who recalled their six months prior hospitalization weight, 44 (35%) presented a weight loss greater than 20% and 70 (55%) presented a weight loss greater than 10%. Overall, malnutrition (BMI<18.5 kg/m^2^) was found in 55 (43%) of the patients and severe malnutrition (BMI <16 kg/m^2^) in 19 (15%). Lean body mass and fat body mass were lower than the 5^th^ percentile of a reference population for 80 (63%) and 38 (30%) patients, respectively. A majority of the patients had anemia (median hemoglobin 10.2; IQR 9.1–12.0 mg/dL) and hypoalbumenemia (median 2.4; IQR 1.8–2.9 g/dL).

**Table 2:** Nutritional characteristics of patients hospitalized with AIDS according to the method used to determine weight and height.

Characteristic		All patients (N = 127)	Method for determining weight and height*	
			Measured (n = 98)	Estimated (n = 29)
		Number (%) or median [IQR]		
Weight loss (%)†	Severe (>20.0)	44 (35)	32 (33)	12 (42)
	Moderate (10.1–20.0)	26 (20)	25 (26)	1 (3)
	Mild (≤10.0)	21 (17)	20 (20)	1 (3)
	None	36 (28)	21 (21)	15 (52)
Body mass index (kg/m^2^)	<16.00	19 (15)	13 (13)	6 (21)
	16.00–16.99	10 (8)	10 (10)	0 (0)
	17.00–18.49	26 (20)	19 (19)	7 (24)
	18.50–24.99	68 (54)	53 (54)	15 (52)
	≥25.00	4 (3)	3 (3)	1 (3)
Lean body mass depletion‡	Severe	80 (63)	64 (65)	16 (55)
	Mild to moderate	20 (16)	13 (13)	7 (24)
	Within normal limits	27 (21)	21 (21)	6 (21)
Body fat mass depletion§	Severe	38 (30)	30 (31)	8 (28)
	Mild to moderate	47 (37)	39 (40)	8 (28)
	Within normal limits	42 (33)	29 (30)	13 (45)
Albumin (g/dL)¶		2.4 [1.8–2.9]	2.4 [1.8–2.9]	2.3 [2.1–2.8]
Hemoglobin (mg/dL)		10.2 [9.1–12.0]	10.1 [8.7–11.8]	10.9 [9.2–12.7]

* Measured = height and weight were directly measured. Estimated = height and weight were estimated [20], [21]. None of the differences between the two groups was statistically significant at α = 0.05.

† Percentage of body weight loss was estimated based on the weight at hospital admission and the patient’s self-reported weight of six months prior to current illness.

‡ Lean body mass depletion was estimated using mid-upper arm muscle area with a correction for the bone area. Categories for lean body mass depletion were defined by comparison of the estimator to population norms [26]. Depletion was classified as within normal limits (>15^th^ percentile), mild to moderate depletion (5^th^–15^th^ percentiles) and severe depletion (<5^th^ percentile).

§ Body fat mass depletion was estimated using triceps skinfold thickness. Categories for body fat mass depletion were defined by comparison of the estimator to population norms [26]. Depletion was classified as within normal limits (>15^th^ percentile), mild to moderate depletion (5^th^–15^th^ percentiles) and severe depletion (<5^th^ percentile).

¶ Albumin values were available for 105 patients (82 and 23 with weight and height measured and estimate, respectively).

IQR = interquartile range.

### Correlates of Malnutrition at Hospitalization

Table 3 summarizes the findings of univariate and multivariable analyses associating patient characteristics with malnutrition at hospital admission. Patients with malnutrition were older and had lower *per capita* household income in comparison to those without malnutrition. Sex, disease duration, the degree of immune suppression, and drug or alcohol use did not differ significantly between those with and without malnutrition. Chronic diarrhea at admission was the only clinical diagnosis associated with malnutrition in univariate analyses.

**Table 3:** Patient characteristics associated with malnutrition (BMI <18.5 kg/m^2^) at hospitalization among patients with AIDS.

Characteristic		BMI <18.5 kg/m^2^ (N = 55)		BMI ≥18.5 kg/m^2^ (N = 72)		Unadjusted PR (95% CI)	Adjusted PR (95% CI)
		n	Number (%) ormedian [IQR]	n	Number (%) ormedian [IQR]		
Male sex		55	29 (53)	72	49 (68)	0.70 (0.47–1.04)	
Age (years)		55	39 [31]–[45]	72	33 [29]–[42]	1.02 (1.00–1.04)	1.02 (1.00–1.04)
Formal education (years)		55	6 (4–11)	72	7 (5–12)	0.98 (0.94–1.02)	
Formally employed		55	8 (15)	72	12 (17)	0.91 (0.51–1.62)	
*Per capita* household income (USD/day)	< $2.00	55	16 (29)	72	12 (17)	2.29 (1.07–4.91)	2.01 (1.06–3.81)
	$2.00–$4.99		18 (33)		23 (32)	1.76 (0.81–3.81)	1.75 (0.92–3.35)
	$5.00–$9.99		15 (27)		19 (26)	1.76 (0.80–3.89)	1.42 (0.76–2.65)
	≥ $10.00		6 (11)		18 (25)	1.00	1.00
Participant of cash payments program*		55	18 (33)	72	17 (24)	1.26 (0.84–1.90)	
Current or prior HAART use		55	26 (47)	71	32 (45)	0.95 (0.64–1.41)	
Time from HIV disease to current hospitalization†	At hospitalization‡	55	16 (29)	70	24 (34)	1.00	
	≤2 years prior		16 (29)		20 (29)	1.11 (0.66–1.88)	
	3–10 years prior		16 (29)		20 (29)	1.11 (0.66–1.88)	
	≥11 years prior		7 (13)		6 (9)	1.35 (0.72–2.53)	
CD4 count <200 cells/mm^3^		43	32 (74)	57	41 (72)	1.08 (0.64–1.82)	
Chronic diarrhea (>30 days)‡		54	29 (54)	72	23 (32)	1.65 (1.11–2.46)	1.42 (0.99–2.04)
Pulmonary tuberculosis‡		54	17 (31)	72	17 (24)	1.24 (0.82–1.89)	

* Self-reported participant of a direct cash payments program (*bolsa família*) from the Brazilian government as part of a national effort to reduce severe poverty and food insecurity.

† Represents the length of time the patient was aware of diagnosis of HIV disease prior to current hospitalization.

‡ Diagnosis made at current hospitalization.

IQR = interquartile range.

BMI = body mass index (kg/m^2^).

PR = prevalence ratio.

CI = confidence interval.

USD = United States dollar.

HAART = highly active antiretroviral therapy.

Multivariable analyses identified older age (2% [95% CI 0–4%] increase in the prevalence of malnutrition for each additional year of age) and very low *per capita* household income as patient attributes independently associated with malnutrition. Living with a daily *per capita* household income of less than USD 2.00, USD 2.00–4.99 or USD 5.00–9.99 increased the prevalence of malnutrition by 2.01 (95% CI 1.06–3.81), 1.75 (95% CI 0.92–3.35) and 1.42 (95% CI 0.76–2.65) times, respectively, compared to the patients whose *per capita* household income was USD 10.00 per day or greater. Diagnosis of chronic diarrhea at admission was marginally associated with malnutrition (PR 1.42; 95% CI 0.99–2.04) and was kept in the final model because it improved its fitness.

### Malnutrition and Death

There was a trend toward increased risk of death among patients who had malnutrition at admission. While 11 (22%) of the 50 patients with malnutrition and available data on outcome died, 8 (12%) of the 68 patients without malnutrition died (RR 1.87; 95% CI 0.81–4.31; chi square P = 0.14).

## Discussion

The Brazilian National STD/AIDS Program is recognized worldwide as a successful example of a nationally integrated HIV/AIDS prevention, medical care, and antiretroviral treatment strategy. From 1996, when HAART was introduced and guaranteed free of cost to every Brazilian in need, the number of patients receiving HAART continuously increased in Brazil, reaching around 200,000 patients with top-of-the-line antiretroviral drugs in 2010 [28]. AIDS incidence subsequently stabilized and mortality declined [29], [30]. As a consequence, the wasting syndrome has become a less frequent clinical concern and the nutritional management of patients with AIDS has been largely directed to the lipodystrophy and metabolic alterations associated with HAART [31], [32]. This study is one of the first in Brazil to describe the prevalence of nutritional deficiencies in hospitalized patients in the era of HAART. Cross-sectional studies have shown the prevalence of malnutrition in Brazilian outpatients with HIV to be 3–6% [33]–[35]. Our findings show that malnutrition remains an important public health problem among patients hospitalized with AIDS, affecting 43% of those admitted to the public reference hospital for infectious diseases in Brazil’s third largest city. In fact, our results may underestimate the burden of malnutrition at hospitalization because we did not include patients with more severe disease presentation, such as those with cognitive impairment and immediate intensive care requirement.

Reasons for AIDS-related hospitalization are diverse and may include late diagnosis, non-adherence or poor access to HAART, and clinical failure of treatment. Our results showed that one-third of our patients were first informed of their HIV disease during the current hospitalization and a large proportion of those with known HIV disease at admission had been hospitalized previously. Although a majority of the patients who knew about their HIV infection before hospitalization reported prior use of HAART, most HAART users reported at least one recent interruption in therapy. These results suggest that strategic improvements to Brazil’s national AIDS program need to address early testing and treatment adherence among marginalized populations.

Older age and daily *per capita* household income <USD 2.00 were independently associated with malnutrition at hospitalization. We also found that prior or current HAART use was statistically associated with higher CD4 cell count and lower viral load, but neither HAART use nor CD4 cell count was associated with malnutrition. These findings may be due to the fact that this patient population had both inconsistent HAART use and a median CD4 cell count below a level protective against malnutrition. Additionally, we did not find an association between malnutrition and tuberculosis, which was identified in one-third of the patients in our study. Malnutrition in HIV-infected persons is a well-described risk for reactivation and primary progression of tuberculosis, and conversely, tuberculosis itself may result in malnutrition [36], [37]. The absence of an association between CD4 cell count, prior or current HAART use, and certain medical conditions (e.g., tuberculosis) with malnutrition suggests that the effect of poverty on nutritional status in HIV may not necessarily be mediated by comorbidities. The mechanisms by which poverty increases the likelihood of malnutrition in HIV and non-HIV populations have been studied in other settings [38], [39]. It is critical that future studies elucidate the mechanisms between poverty and malnutrition in Brazil and advance our understanding of how to develop effective interventions.

We found an increased prevalence of malnutrition among patients with chronic diarrhea in the univariate analysis. This association might have achieved statistical significance in the multivariable analysis if we had a larger patient sample. While access to effective antiretroviral therapy remains the foundation of HIV treatment strategies, our results suggest that AIDS-related morbidity may be further reduced by renewed attention to chronic diarrhea as a clinical condition that contributes to malnutrition. HIV-related enteropathy reduces the immunologic capacity of the gastrointestinal tract and results in villous atrophy [40], which leads to diarrhea and malabsorption. This process can be further aggravated by opportunistic enteric pathogens [41].

This study was not designed to characterize the relationship between malnutrition and the requirement for hospitalization in patients with AIDS, nor was it developed to evaluate the impact of malnutrition on the risk of death. We nonetheless identified a trend toward a higher in-hospital case fatality ratio among patients with malnutrition. This finding is in accordance with current evidence about the importance of good nutrition on survival of patients with HIV [42], [43]. Future studies are needed to determine the best nutritional interventions to decrease the burden of deaths in malnourished patients with advanced HIV disease.

Three public hospitals each accounted for approximately one-third of the AIDS hospitalizations in Salvador during 2009–2010: the study hospital (32% of hospitalizations), a state general hospital (33%) and a university hospital (31%) [16]. All three provided specialized ambulatory AIDS care during the study period. However, only the study hospital and the state general hospital provided 24-hour open-door emergency services. Because patients may either be referred for hospitalization by providers or they may self-present to hospital, those admitted to the study hospital and to the state general hospital are probably more representative of patients with AIDS requiring inpatient care in Salvador. While the study population is probably similar to those at other open-door public hospitals in Brazil, it may not represent AIDS-related admissions throughout the country, particularly those at private institutions.

This study has other limitations. We relied on patient recall for retrospective ascertainment of clinical data, including the patient’s weight six months prior to hospitalization and the year of first patient knowledge of HIV disease. Some patients may have confused the distinction between an initial notification of HIV infection and a subsequent AIDS diagnosis. Further, given the delay in diagnosis seen in this patient population, this variable probably does not accurately reflect duration of HIV disease. We applied anthropometric measurements to define body fat and muscle mass. Anthropometrics are rough indicators of body composition and are less precise than other methods (e.g., bioelectrical impedance). However, they are sufficiently accurate for assessing the public health burden of malnutrition [44], as was the aim of this study. Lastly, for bed-bound participants we estimated height and weight to calculate BMI, which could have misclassified some patients by nutritional status.

Our patient population demonstrated a high level of malnutrition and weight loss at hospital admission in a country long considered to be an international model for HIV care. These results point to substantially unmet nutritional needs for a sizeable group of Brazilians hospitalized with AIDS. They should further reinforce for clinicians the importance of performing nutritional evaluations and simple body composition studies in all patients with HIV [45], [46], as malnutrition is a modifiable predictor of death in these individuals [4]–[7]. Improving early testing and HAART adherence strategies, especially for vulnerable populations, may continue to help reduce AIDS-related morbidity and mortality in Brazil. It is nonetheless also critical to identify new methods for interrupting the cycle of poverty, HIV, and malnutrition.
